# Book Reviews

**Published:** 1892-01

**Authors:** 


### Book Reviews.

PRACTICAL PATHOLOGY AND MORBID HISTOLOGY. By HENEAGE GIBBES, M. D., Professor of Pathology in the University of Michigan; formerly Lecturer on Normal and Morbid Histology in the Medical School of the Westminster Hospital, London; formerly Curator of the Anatomical Museum, King's College, London. Illustrated with sixty photographic reproductions. Octavo, pp. xii.—320. Philadelphia: Lea Brothers & Company. 1891.

A new work on Pathology by an American author is always hailed with interest and pleasure. Such was the feeling when Professor Gibbes's work first appeared. The original researches, which have been instituted by him within the past few years, have given his name a prominence in this field of medicine. We regret that this work is intended for laboratory students, instead of being an exhaustive treatise on the subject.

The first seven chapters are devoted to the preparation of the material, and is a careful and painstaking description of the different processes of mounting, staining, etc. We, however, question the advisability of incorporating these chapters in a work on pathology, because, with but little exception, they are the same as are in vogue in normal histology, and no student should study pathology before he knows microscopy and histology quite thoroughly.

Chapters VIII. to XIII. are devoted to the study of Bacteriology, and here again we feel as if the subject of pathology must suffer by just so much. Today bacteriology is regarded as so separate a branch, that it seems out of place in a work on pathology.

In chapter XIV. the real subject is taken up. The degenerations and inflammations deserve, perhaps, more attention than they have received; still, as discussed, they must convey to the reader a good knowledge of the changes which they provoke in the system. The tumors are next taken up, the fibromata and sarcomata being exceptionally well presented. Most authors pass by hurriedly the different forms of fibroma, such as fibrous epulis, nasal and rectal polypi, etc., but not so in this work.

The chapters on Diseases of the Heart, and Nervous System, are treated much too sparingly, even for students; whereas, the chapters on Diseases of the Lungs and Arteries deserve special commendation. The chapters on the Liver, Kidney, Spleen, Tongue, etc., are briefly but ably discussed.

Part IV., chapters XLI. to XLIV., are devoted to photography with the microscope, and here again the methods and formula are fully and thoroughly described. The illustrations, sixty in number, are all micro-photographs. Some of them are great aids, but some convey little meaning, especially the illustration of a spindle-celled sarcoma, page 165, and the melanotic sarcoma, page 169. Until we can photograph colors, thereby distinguishing between a cell body and cell nucleus, micro-photography will be of little service in microscopical pathology.

The publishers, as usual, have contributed their full share towards making it a pleasing and desirable work. W. C. K.

---

THERAPEUTICS: ITS PRINCIPLES AND PRACTICE. By H. C. WOOD, M. D., LL.D., Professor of Materia Medica and Therapeutics, and Clinical Professor of Diseases of the Nervous System, in the University of Pennsylvania. A work of Medical Agencies, Drugs and Poisons, with especial reference to the relations between Physiology and Clinical Medicine The eighth edition of Treatise on Therapeutics rearranged, rewritten, and enlarged. Octavo, pp. 937. Philadelphia: J. B. Lippincott Company. 1891.

It is so recently (October, 1888,) that we had occasion to speak in these columns in reference to the value of Dr. Wood's Treatise on Therapeutics, when we reviewed the seventh edition, it seems almost a marvel to find ourselves now called upon to mention the eighth edition of this matchless work. The fact that another edition is called for so soon, will serve, in a certain degree, to mark the rapidity of movement that is making in the science of therapeutics as applied to the treatment of disease. Dr. Wood has made himself so distinctly an authority on this subject, that no one presumes to question the fact that Wood's Therapeutics is rapidly becoming an American classic. In the present edition the author has brought forward everything pertaining to his subject to the latest date, and, taken altogether, it is one of the most complete single volume treatises on therapeutics extant.

This edition has been thoroughly revised, and some of the work is practically new. For instance, the whole subject of Anesthetics, the articles upon Cocaine, Strophanthus, Caffeine, Antipyrin, Antifebrin, Phenacetin, Hydrastin, Paraldehyd, Lead-poisoning, etc., have been rewritten ; while the articles on Sulphonal, Chloralamid, Aristol, and some others, are entirely new.

We recommend every student of medicine to obtain Wood's Therapeutics, and every practitioner who would freshen up his library, should do likewise.

The publishers are entitled to high commendation for the part they have played in putting forth this new edition so promptly and handsomely.

---

A Short Manual of Analytical Chemistry, Qualitative and Quantitative, Inorganic and Organic. Arranged on the principle of the course of instruction given at the South London School of Pharmacy. By John Muter, M. A., Ph. D., F. R. S. E., F. I. C., F. C. S., Analyst to the Metropolitan Asylum Board ; Public Analyst for Lambeth, Wansworth, Southwark, Newington, Rotherhithe, and the Linsey Division of Lincolnshire ; Past President of the Society of Public Analysis ; author of Pharmaceutical and Medical Chemistry (Theoretical and Descriptive), A Key to Organic Materia Medica, late editor of *The Analyst*, etc., etc. First American from the fourth English edition, edited by Claude C. Hamilton, M. D., Ph. G., Professor of Analytical Chemistry in the University Medical College and the Kansas City College of Pharmacy ; Professor of Chemistry in the Western Dental College of Kansas, Missouri. Octavo, pp. 205. Philadelphia : P. Blakiston, Son & Co., 1012 Walnut street. 1891.

This volume, which comes from the pen of a public and private analyst of wide reputation, is an excellent one, and contains within its two covers a great deal of valuable information. Although primarily intended for students, it is of value to the busy analyst as well, for the methods are given in clear and concise manner, thus rendering a saving of time, which is further increased by an excellently arranged index.

We wish to call the editor's attention to what we believe is an oversight. On page 166, under the heading Fat, the author says: "The following method of estimating fat is generally followed by most of the official milk analysts of America: Replace the dish containing the total solids upon the water bath, and fill from a wash bottle with petroleum naphtha of the quality of benzine (U. S. P., 1880). Allow the benzine to boil down to about one-half, and decant against a rod into a basin, replace upon the water bath, and again refill with naphtha, and decant. Repeat this four times, then wash the outside of the capsule with benzine, dry the dish, and weigh as in total solids. The loss of weight of the total solids is the fat in 5 c.c. of milk taken." We do not believe that the best analysts of this country use this method today. At all events it is not a method which would stand much show in a court of law, and official analysts do, or always should, bear in mind that methods may be questioned by attorneys.

From the reference (U. S. P., 1880,) we judge that this was taken from the U. S. P., which is a decidedly unreliable book from which to get the analytical methods of today.

The typography and general make-up of this volume is in the usual excellent style and good taste so characteristic of these publishers. J. A. M.

---

A Clinical Text-Book of Medical Diagnosis for Physicians, based on the Most Recent Methods of Examination. By Oswald Vierordt, M. D., Professor of Medicine at the University of Heidelberg; formerly Privat-Docent of the University of Leipzig; later Professor of Medicine and Director of the Medical Polyclinic of Jena. Authorized translation from the second improved and enlarged German edition, with additions. By Francis H. Stuart, A. M., M. D., New York. With 178 illustrations, many of which are in colors. Octavo, pp. xvi. — 700. Philadelphia: W. B. Saunders. 1891. Price, cloth, $4.00 net; sheep, $5.00 net.

The subject of medical diagnosis is one always fraught with interest, and its importance is concededly of the first. The work before us is written by a man of experience and judgment, and it cannot fail to command the attention of the American medical profession. It is difficult for any author on this subject to gain a footing in the schools as against DaCosta, who has held sway so long, but we are of the opinion that Vierordt has constituted himself a successful rival, whose treatise will compete for the honor of being assigned as a text-book in the American college courses.

This work possesses the merit of being entirely abreast of modern science in every respect, as applied to the diagnosis of disease. It is illustrated with clear cuts in wood and in colors, which elucidate the author's meaning in an admirable manner. No student can be considered equipped for the final examination until he has mastered questions of diagnosis, and no physician is competent to treat disease until he is thoroughly skilled in the principles and technique of differential and absolute diagnosis of disease. Vierordt classifies his treatise in a systematic and business-like way. He divides his subjects into three general parts. In Part I. he gives an introduction and a chapter on the Examination of Patients; in Part II., general examination is considered; in Part III., the special examination of the several tracts, apparatuses and systems of the viscera are discoursed upon. His sentences are clear, epigrammatical, and finished. His style is that of a scholar who knows what he is writing about, says what he has to say, and stops without a superfluous word or sentence. The consequence is that into this work there is crowded an immense amount of material such as cannot be found in any treatise in the corresponding space. The index is a copious one, indeed, one of the best we have ever seen, which comprises about ninety pages of the volume. We advise every student of medicine to possess this work with little delay, and every practitioner would do wisely if he should add it to his library. The publisher has presented it in a most attractive form.

---

THE COMPARATIVE ANATOMY OF THE DOMESTICATED ANIMALS. By A. CHAUVEAU, M. D., LL. D., Member of the Institute (Academy of Sciences); Inspector-General of Veterinary Schools in France; Professor of the Museum of Natural History, Paris. Revised and enlarged, with the coöperation of S. Arloing, Director of the Lyons Veterinary School, Professor of Experimental and Comparative Medicine at the Lyons Faculty of Medicine. Second English edition, translated and edited by George Fleming, C. B., LL. D., F. R. C. V. S., Late Principal Veterinary Surgeon of the British Army; Foreign Corresponding Member of Société Royal de Medicine, and the Société Royal de Medicine Publique, of Belgium; Foreign Associate of the Société Centrale de Medicine Veterinaire of France; Honorary Life Member of the Royal Agricultural Society of England; Foreign Member of the Société Nationale d' Agriculture of France, etc.; Examiner in Anatomy for the Royal College of Veterinary Surgeons. With 585 illustrations. New York: D. Appleton & Co. 1891.

The scope of this valuable work is limited to what the author calls veterinary anatomy; but the veterinary surgeon is not the only one to receive the benefit of its teachings. Even the medical man, who has not given time to the study of comparative anatomy in its more comprehensive form, may find much in its pages to interest him. Founded on human anatomy, from which its nomenclature is largely borrowed, all that is necessary to enable one not familiar with this science is clearly given both in text and illustrations, to enable him to follow the author, so as to get a very clear idea of the analogies and differentials between the lower animals, of whose structure and histology he treats.

At the outset we have an enumeration and classification of domesticated animals, general ideas of their organization and description of the various cells and tissues which go to make up the framework and physiology of a domestic animal, with all the apparatuses necessary to its life and well-being. The bony framework of the various classes is well illustrated and described, and the differentiations between them, as well as their general similarity of plan to the human body, are clearly seen at a glance. So of the muscular arrangement and adaptation throughout the whole range, from the camel to the domestic fowl.

Such a work as this cannot fail to interest all who are interested in domestic animals; and who is not? Of course, to the veterinary doctor it is a *sine qua non*, and no painter or sculptor of animal life can attain his highest ideal without familiarity with the science as recorded in its pages.

Too much cannot be said in praise of the beauty and adaptation of its 585 illustrations that run through its 1,050 pages. A very copious index gives the book additional value.

---

THE MICROSCOPE AND HISTOLOGY, PART I. The Microscope and Microscopical Methods. By SIMON HENRY GAGE, Associate Professor of Physiology, Cornell University, Ithaca, New York, U. S. A. Pp. VIII. + VI. + 96; five full page plates and eight figures in the text. Ithaca, N. Y.: Andrus & Church. 1891. Price, bound in heavy manilla, $1.00; half leather, $1.25.

Part I. of Professor Gage's prospective work on The Microscope and Histology, is now put upon the market in a new and convenient form. Originally intended for his students at Cornell University, it has outgrown those limits, and we bespeak for it a large and appreciative field. We know of no work on microscopy that so thoroughly and effectively discusses the principles and practice of this important science as Prof. Gage's book. The attention and scrutiny paid to details in the elements of a science, so characteristic of the Cornell school—thus laying a broad and solid foundation for the superstructure—cannot help but make students scientists before they leave the college halls. Prof. Gage has collected material, especially the tables on Tube-Lengths, Cover-Glass Thickness, etc., of great value to the microscopists, which can be found in no other work on this subject. To the student or physician who wishes to study the microscope and methods, we can heartily recommend this work, and feel assured that the knowledge thus gained will be of inestimable value in pursuing the higher sciences, as histology, pathology, and bacteriology. We await with pleasure the appearance of Part II. on Histology.

Much credit is due Mrs. Professor Gage for the richly executed plates and drawings, which add materially to the value of the work.

Messrs. Andrus & Church have performed their work very satisfactorily, and deserve to be better known as publishers of scientific works. W. C. K.

---

Essentials of Bacteriology: Being a Concise and Systematic Introduction to the Study of Microörganisms for the use of Students and Practitioners. By M. V. Ball, M. D., late Resident Physician German Hospital, Philadelphia; Assistant in Microscopy, Niagara University, Buffalo, N. Y., etc. With seventy-seven illustrations, some in colors. Saunders' Question Compends, No. 20. Philadelphia: W. B. Saunders, 913 Walnut street. 1891.

As one of our exchanges has remarked, the "compends are the fruit of the two years' courses," but if we must have compends, we prefer to have them as complete and thorough as the word compend will allow. The book by Dr. Ball is an excellent little work, as far as it goes, and is on a level, if not superior, to those which have appeared in this series. The arrangement is admirable, and the many cuts, some in colors, add materially to its instructiveness. As an aid or guide for laboratory students already versed in bacteriology, it has its greatest value. W. C. K.

---

Practical Notes on Urinary Analysis. By William B. Canfield, A. M., M. D., Chief of Chest Clinic and Lecturer on Clinical Medicine, University of Maryland; Visiting Physician to the Union Protestant Infirmary, Bay View Hospital, Baltimore, etc. Physicians' Leisure Library, Detroit, Mich.: George S. Davis. 1891. Price, cloth, 50 cents; paper, 25 cents.

In this compact little book Dr. Canfield has grouped a vast amount of useful information in convenient form. It is on a subject of sufficient importance for every professional man to possess the book, and its cheapness brings it within the range of everybody's purse. We observe that Dr. Canfield insists upon spelling albumin in the old way, with an "e," and yet he uses an "i" when he converts it into albuminuria. The last chapter in the book on the Order of Analysis is a very compact and useful presentation of a system that should prevail in every urinary analysis, and it is especially commended to examiners of life insurance as a guide.

---

A MANUAL OF PRACTICAL OBSTETRICS. By EDWARD P. DAVIS, A. M., M. D., Clinical Lecturer on Obstetrics in the Jefferson Medical College, Professor of Obstetrics and Diseases of Children in the Philadelphia Polyclinic, etc.; with 140 illustrations, two of which are colored. Philadelphia: P. Blakiston, Son & Co., 1012 Walnut street. 1891.

The author states in the preface that the preparation of this book has been suggested by the needs experienced in teaching students of medicine, and he has incorporated such scientific facts as would meet the demands of the practitioner who requires the science so rendered that they are presented at a glance. The works of Parvin, Lusk, Schröder, Muckel, Carl Braun, Galabin and Dühussen have been used in the preparation of the book, thus aiming to furnish the latest and best thought of authorities resorted to by the profession. The author has succeeded well in this task, and presents a valuable little manual, which will subserve an excellent purpose for the student and practitioner, who have not the time to examine the larger works. In carefully perusing its pages, we are surprised that so much of the science and art of obstetrics can be presented in so small a compass. The book is one of the most practical of its kind to which our attention has been called.

---

ARTIFICIAL ANESTHESIA AND ANESTHETICS. By DEFOREST WILLARD, A. M., M. D., Ph. D., Clinical Professor of Orthopedic Surgery in the University of Philadelphia; Surgeon to the Presbyterian Hospital, etc., and LEWIS H. ADLER, JR., M. D., Instructor in Rectal Diseases, Philadelphia Polyclinic and College for Graduates in Medicine. The Physicians' Leisure Library. Single copies, cloth, 50 cents; paper, 25 cents. Detroit, Mich.: Geo. S. Davis. 1891.

Every physician, no matter what his special line of practice, should thoroughly familiarize himself with the administration of anesthetics. With the constantly increasing necessity for their use both in medicine and surgery, it is important that all possible safeguards should be thrown around the administration of any drug which possesses local or general anesthetic effects. If every physician in the land would read this little work of Drs. Willard and Adler, they would certainly be better equipped for dealing with emergencies that may arise in the use of these important aids to the successful practice of medicine and surgery. Particularly do we commend the chapter on the Medico-legal Relations of Artificial Anesthesia, which is the last one in the book.

---

Essentials of Physiology, Arranged in the Form of Questions and Answers, Prepared Especially for Students of Medicine. By H. A. Hare, B. Sc., M. D., Professor of Therapeutics and Materia Medica in the Jefferson Medical College of Philadelphia; Physician to St. Agnes' Hospital, and to the Medical Dispensary of the Children's Hospital; Laureate of the Royal Academy of Medicine in Belgium, of the Medical Society of London, etc.; Secretary of the convention for the revision of the Pharmacopeia. 1890. Saunders' Question-Compends, No. 1. Third edition, thoroughly revised and enlarged by the addition of a series of handsome plate illustrations taken from the celebrated Icones Nervorum Capitis of Arnold. Philadelphia: W. B. Saunders, 913 Walnut street. 1891.

In this volume of question compends we find the subject of physiology concisely and accurately considered. It will be found an aid to the memory in mastering the subject, but should not be in any sense taken to supplant the general treatise or to prevent the efficient laboratory work in acquiring a knowledge of one of the fundamental departments of medicine. Dr. Hare has presented the subject in the best light possible for this particular form of literature.

---

The Surgical Treatment of Wounds and Obstruction of the Intestines. By Edward Martin, M. D., Instructor in Operative Surgery, University of Pennsylvania, Surgeon to the Howard Hospital, Assistant Surgeon to the University Hospital; and H. A. Hare, M. D., Professor of Therapeutics, Jefferson Medical College; Attending Physician to St. Agnes' Hospital. Fiske Fund Prize Dissertation. No. XL., 8vo, pp. 169. Philadelphia: W. B. Saunders. 1891. Price, cloth, $2.00 net.

The importance attaching to wounds and obstructions of the intestines makes it fitting and proper that a monograph should be prepared on these subjects, which shall consider them from both the medical and surgical sides of the question. Drs. Martin and Hare have presented such a monograph in the work under consideration, and it is one which deserves the careful study of physicians, who either practice on the one side or the other of the question. In this book, consisting of an introduction, fourteen chapters, and a table of cases, together with an index, will be found some of the most modern thought pertaining to the important subjects considered, that commends it to the profession as an able exposition thereof.

---

A COMPEND OF HUMAN PHYSIOLOGY, Especially Adapted for the Use of Medical Students. By ALBERT P. BRUBAKER, M. D., Professor of Physiology, Pennsylvania College of Dental Surgery; Demonstrator of Physiology in Jefferson Medical College, Philadelphia. Sixth edition; Revised and improved, with new illustrations. Quiz Compends No. 4. Philadelphia: P. Blakiston, Son & Co. 1891.

Considering the fact that Dr. Brubaker's little book has passed to its sixth edition, there is no question as to its having become a standard of its kind. It is as good a compilation of the subject as has yet appeared, and will, no doubt, command ready sale.

---

THREE THOUSAND QUESTIONS ON MEDICAL SUBJECTS, arranged for Self-Examination, with the proper references to standard works, in which the correct replies will be found. Philadelphia: P. Blakiston, Son & Co. 1891.

These questions have been admirably arranged with reference to the object desired to be attained. The frequent self-examination of a student may be very greatly systematized through a work of this kind, but we cannot see any essential reason why it should be applied to any special works or references. It is intended by the publishers to present the book gratis to any who will apply for it, enclosing ten cents for postage.

---

MODERN MATERIA MEDICA FOR PHARMACISTS, MEDICAL MEN AND STUDENTS. By H. HELBING, F. C. S. Second enlarged edition. Cloth, 115 pages, 75 cents. Published by the *British and Colonial Druggist*. London: Lehn & Fink, New York, sole agents for the United States.

This little work, intended as a help to pharmacists and medical men, is the first detailed scientific treatise on new remedies published in the English language. It not only elaborates fully upon the constitution, methods of preparation, processes for identification and testing of the various substances, but gives valuable hints as to the most approved methods of prescribing and dispensing these various synthetic remedies. A valuable appendix, including tables of doses, melting and boiling points, and solubilities, is added.

The author has given us a very valuable work, and one which will be thoroughly appreciated by professional men. We recommend it most heartily to the busy pharmacist and physician.

